# Safety and Immunogenicity Following the Second and Third Doses of the BNT162b2 mRNA COVID-19 Vaccine in Adolescents with Juvenile-Onset Autoimmune Inflammatory Rheumatic Diseases: A Prospective Multicentre Study

**DOI:** 10.3390/vaccines11040819

**Published:** 2023-04-10

**Authors:** Merav Heshin-Bekenstein, Amit Ziv, Natasa Toplak, Siman Lazauskas, Danielle Kadishevich, Efrat Ben-Nun Yaari, Adi Miller-Barmak, Yonatan Butbul Aviel, Esther Saiag, Sara Pel, Ori Elkayam, Yosef Uziel, Victoria Furer

**Affiliations:** 1 Pediatric Rheumatology Service, Dana-Dwek Children’s Hospital, Tel Aviv Sourasky Medical Center, Tel Aviv 6423906, Israel; 2 Sackler Faculty of Medicine, Tel Aviv University, Tel Aviv 6997801, Israel; 3 Pediatric Rheumatology Unit, Meir Medical Center, Kfar Saba 4428164, Israel; 4 Department of Allergology, Rheumatology and Clinical Immunology, University Children’s Hospital, University Medical Center Ljubljana, 1000 Ljubljana, Slovenia; 5 Faculty of Medicine, University of Ljubljana, 1000 Ljubljana, Slovenia; 6 Pediatric Department, Dana-Dwek Children’s Hospital, Tel Aviv Sourasky Medical Center, Tel Aviv 6423906, Israel; 7 Pediatric Department, Meir Medical Center, Kfar Saba 4428164, Israel; 8 Pediatric Rheumatology Unit, Rambam Medical Center, Haifa 3109601, Israel; 9 Hospital Management, Information and Operation Branch, Tel Aviv Sourasky Medical Center, Tel Aviv 6997801, Israel; 10 Rheumatology Department, Tel Aviv Sourasky Medical Center, Tel Aviv 6423906, Israel

**Keywords:** vaccines, COVID-19, pediatric rheumatology, juvenile-onset rheumatic diseases, safety, immunomodulatory medications, biologics, adolescent

## Abstract

Background: To explore the long-term safety and dynamics of the immune response induced by the second and third doses of the BNT162b2 mRNA COVID-19 vaccine in adolescents with juvenile-onset autoimmune inflammatory rheumatic diseases (AIIRDs) compared with healthy controls. Methods: This international prospective study included adolescents with AIIRDs and controls vaccinated with two (AIIRDs n = 124; controls n = 80) or three (AIIRDs n = 64; controls n = 30) doses of the BNT162b2 vaccine, evaluated for vaccine side-effects, disease activity, COVID-19 breakthrough infection rates and severity, and anti-spike S1/S2 IgG antibody titers in a sample from both groups. Results: The vaccination safety profile was favorable, with most patients reporting mild or no side-effects. The rheumatic disease remained stable at 98% and 100% after the second and third doses, respectively. The two-dose vaccine induced comparable seropositivity rates among patients (91%) and controls (100%), (*p* = 0.55), which declined within 6 months to 87% and 100%, respectively (*p* = 0.3) and increased to 100% in both groups after the third vaccine dose. The overall post-vaccination COVID-19 infection rate was comparable between patients and controls, 47.6% (n = 59) and 35% (n = 28), respectively; *p* = 0.5278, with most infections occurring during the Omicron surge. In relation to the last vaccination, time-to-COVID-19 infection was similar between patients and controls, at a median of 5.5 vs. 5.2 months, respectively (log-rank *p* = 0.1555). Conclusion: The safety profile of three doses of the BNT162b2 mRNA vaccine was excellent, with adequate humoral response and similar efficacy among patients and controls. These results support the recommendation for vaccinating adolescents with juvenile-onset AIIRDs against COVID-19.

## 1. Introduction

Patients with juvenile-onset autoimmune inflammatory rheumatic diseases (AIIRDs) on immunomodulatory treatment are prone to infections, including SARS-CoV-2 infection [1]. Thus far, vaccination, along with personal protective measures, have been the mainstay approach to preventing COVID-19 infection [2].

Studies on adults with AIIRDs demonstrated that the two-dose BNTb262 regimen was associated with comparable immunogenicity in patients and healthy controls. Most patients developed an adequate immunogenic response to the vaccines, except for patients on B-cell-depleting therapy [3,4].

Data regarding the safety and immunogenicity of COVID-19 vaccination in adolescents with AIIRDs on immunomodulation was initially lacking, as these individuals were excluded from the vaccine trials. Since 2022, a few studies demonstrated that the two-dose COVID-19 vaccine regimen was safe and immunogenic in adolescents with AIIRDs as compared to healthy adolescents [5,6,7,8].

The waning of vaccine-induced immunity after the two-dose BNT162b2 mRNA vaccine regimen was well-documented [9,10]. In October 2021, the American College of Rheumatology recommended a booster vaccine dose for AIIRD patients receiving any immunosuppressive treatment [11]. This was also supported by the EULAR and PReS recommendations [12,13].

Since then, several studies have shown that the third vaccine dose induces an augmented immunogenic response among patients with AIIRDs [14,15,16,17]. The booster vaccination was also shown to reduce the rates of both confirmed COVID-19 and severe COVID-19 in a large Israeli population of participants ages 60 years or older [18].

However, longitudinal data on the safety and the dynamics of anti-S antibody titer following the second and third doses of the COVID-19 vaccine in adolescents with AIIRDs receiving different immunomodulatory regimens are lacking. Here, we report the results of a multicentre, prospective longitudinal study conducted to investigate the safety and dynamics of the immunogenic response following the second and third doses of the BNT162b2 COVID-19 vaccine in adolescents with AIIRDs and healthy controls.

## 2. Materials and Methods

This international, prospective, multicentre study was conducted from April 2021 to July 2022 at the Pediatric Rheumatology Clinics of Dana-Dwek Children’s Hospital (Tel Aviv), Meir Medical Center (Kfar Saba), and Rambam Medical Center (Haifa) in Israel, and at the University of Ljubljana Children’s Hospital in Slovenia.

### 2.1. Ethical Approval Information

The study was approved by the Institutional Review Board (IRB) of Tel Aviv Sourasky Medical Center, TLV-0175–21 (coordinating center), and by the IRBs of the participating centers. The study was performed in accordance with the principles of the Declaration of Helsinki. Written informed consent was obtained from all study participants.

### 2.2. Study Endpoints

The primary endpoint of the study included the long-term safety of the BNT162b2 mRNA vaccine, as evaluated by side-effects following the second and third doses in adolescents with AIIRDs compared with healthy controls, and the impact of vaccination on the clinical disease activity of the rheumatic disease in the patient group. The secondary endpoints were long-term immunogenicity of the BNT162b2 mRNA vaccine in adolescents with AIIRDs measured at 2–8 weeks and 6 months after the second vaccine and at 2–8 weeks after the third vaccine dose, compared with controls. Breakthrough COVID-19 infections were recorded based on positive SARS-CoV-2 polymerase chain reaction (PCR) tests, documented ≥ 14 days following vaccination.

### 2.3. Study Population

The study population included adolescents (ages 12–18 years) with juvenile-onset of the following AIIRDs: Juvenile Idiopathic Arthritis (JIA) according to the EULAR 2001 classification criteria [19]; systemic lupus erythematosus (SLE) according to the 1997 ACR or 2012 SLICC criteria [20]; systemic vasculitis, i.e., ANCA-associated vasculitis (AAV), including granulomatosis with polyangiitis (GPA), according to the Chapel Hill Consensus Conference definitions [21]; Behçet’s disease, idiopathic uveitis, IBD-related arthritis, systemic or localized scleroderma or juvenile dermatomyositis according to the EULAR/ACR classification criteria [22]; or autoinflammatory syndromes [23,24]. Patients were instructed to continue all medications during the vaccination period. Patients were recruited from four medical centers, including 97 (78%) participants from Israel and 27 (22%) from Slovenia. The control group consisted of healthy adolescent volunteers recruited by Dana Dwek Children’s Hospital, who provided blood samples during a well-being visit. Exclusion criteria were previous COVID-19 infection, and for controls, a history of AIIRDs or immunosuppressive treatment.

All study participants from Israel and Slovenia were vaccinated with two doses of BNT162b2 mRNA vaccine (30 μg per dose, administered intramuscularly, 3 weeks apart). The third vaccine dose was administered at least 6 months after the first vaccine dose, as indicated by the national guidelines.

### 2.4. Safety and Clinical Assessment of the AIIRDs

The participants were contacted by telephone within 2–8 weeks following the second and third vaccine doses and completed a questionnaire regarding local and systemic side-effects.

Medical history and the use of medications were recorded. Immunomodulating medications were continued throughout the study period. Treatment was not withheld before or after the vaccine dose. Data regarding disease activity up to 3 months before vaccination were retrieved from the patients’ medical records. Post-vaccination disease activity was assessed by in-person clinical examination within 3 months after each vaccine dose and at 1-year after the first vaccine.

The following disease activity indices were included: Juvenile Arthritis Disease Activity Score 10 for JIA [25], SLEDAI for SLE, and patients’ and physicians’ global assessments (PGA, PhGA respectively), using a visual analog scale of 0–10 mm, for JIA, SLE, vasculitis, inflammatory myositis, scleroderma, uveitis, and autoinflammatory syndromes.

### 2.5. Vaccine Immunogenicity

Vaccine immunogenicity was evaluated for a subgroup of patients and controls by measuring serum IgG antibody levels against SARS-CoV-2 trimeric spike S1/S2 glycoproteins 2–8 weeks after the second and the third vaccine doses. We used the FDA-authorized LIAISON (DiaSorin, Sallugia, Italy) quantitative assay. The assay provides an indication for the presence of neutralizing IgG antibodies against SARS-CoV-2, and its clinical sensitivity and specificity are >98% [26]. A value of >15 binding antibody units (BAU) was considered positive according to the manufacturer’s instructions.

### 2.6. Breakthrough COVID-19 Infection Rate

Breakthrough infection among fully vaccinated individuals was defined, according to the US Centers for Disease Control and Prevention, as infection occurring ≥ 14 days after the second dose in a two-dose series [27]. Participants were queried for evidence of breakthrough COVID-19 infection following each vaccine dose, and electronic medical records were reviewed for evidence of infection 6 months after each vaccination based on the results of positive SARS-CoV-2 PCR tests. Follow-up was 1 year after the first vaccine dose.

### 2.7. Patient and Public Involvement

The study research question and outcome measures were developed in collaboration with parent representatives of the adolescents with AIIRDs, based on a shared priority to investigate the long-term immune response to the mRNA BNT162b2 vaccine. Parents and patients with AIIRDs under the care of the medical centers conducting the trial were actively informed regarding the study and offered enrolment. The main study results will be disseminated to the parent representatives, who will help in disseminating them through parent networks locally and abroad.

### 2.8. Statistical Analysis

Differences between continuous variables were tested for significance using the independent-sample *t*-test. Differences between categorical variables were tested for significance using the chi-squared test or Fisher’s exact test, each as appropriate. All tests applied were two-tailed, and a *p*-value ≤ 0.05 was considered statistically significant. Survival was analyzed with Kaplan–Meier plots. Median survival durations were reported with 95% confidence intervals (CI). Differences between groups were tested using the Log-Rank test. The data were analyzed using R version 4.0.5 (R Foundation for Statistical Computing, Vienna, Austria).

## 3. Results

### 3.1. Study Sample

A total of 124 adolescents with AIIRDs and 80 healthy adolescents vaccinated with two doses of the BNT162b2 mRNA vaccine participated in the study (Table 1). Among the participants, 64 (54.6%) patients and 30 (37.5%) controls were also vaccinated with the third dose. Both patient and control groups had comparable gender distributions of 60% and 51% females, respectively (*p* = 0.27). Adolescents with AIIRDs were significantly older than controls, mean age of 15.5 years (SD 1.99) compared with 13.7 years (SD 1.36), *p* < 0.0001, respectively. JIA was the most common disease (n = 54, 44%), followed by SLE (n = 20, 16%). The mean disease duration was 4.12 years (SD. 3.79).

Most patients, n = 92 (74%), were receiving immunomodulatory therapies (Table S1). Conventional synthetic DMARDs (csDMARDs) were used by 57 (45.9%) patients, with methotrexate and plaquenil the most frequent (n = 27 (21.8%) and n = 26 (21.1%), respectively), followed by mycophenolate mofetil (MMF) (n = 13, 10.5%). Glucocorticoids were used by 21 (16.9%) patients, mean dose of 11.6 mg/day (SD 11.88). Biologic DMARDs (bDMARDs) were used by 48 (38.7%) patients. TNF inhibitors (TNFi) were the most common bDMARD, used by 39 (31.5%) patients, followed by the IL-6 inhibitor (n = 7, 5.7%) and CD-20 depleting therapy (rituximab (RTX); n = 4, 3.2%). Janus kinase inhibitors were used by five (4%) patients.

### 3.2. Safety of the BNT2b2 Vaccine

The safety profile of the three vaccine doses was favorable, with most participants experiencing either no or mild side-effects (Table 2). Local reactions to vaccination were more common among AIIRD patients compared with controls at 2–8 weeks after the second vaccination, 71% (n = 88) and 55% (n = 44), *p* = 0.0293, respectively, whereas after the third vaccine dose, AIIRD patients had significantly fewer local reactions than controls did, 39% (n = 25) vs. 73% (n = 22), *p* = 0.004, respectively. Following the second vaccine, a few systemic side effects were significantly more prevalent in the AIIRD patients compared to the controls, including chills (14% vs. 4%; *p* = 0.04), fatigue/weakness (27% vs. 15%; *p* = 0.07), myalgia (19% vs. 9%; *p* = 0.08), arthralgia (11% vs. 2%; *p* = 0.04) and headache (23% vs. 11%; *p* = 0.04). Importantly, there were no serious side-effects in either group. No cases of pericarditis or myocarditis were observed. Five patients (4%) experienced an exacerbation of rheumatic disease shortly after the first vaccine dose, and three patients (2%) after the second dose. Two patients, both with AAV, were hospitalized shortly after the first vaccine dose, as previously reported by our group [5]. None of the patients who received the third vaccine reported subsequent rheumatic disease exacerbation or hospitalization. Disease activity remained stable at 1-year clinical follow-up in all patients.

### 3.3. Humoral Response to the BNT162b2 s and Third Vaccine Doses

**Figure 1:**
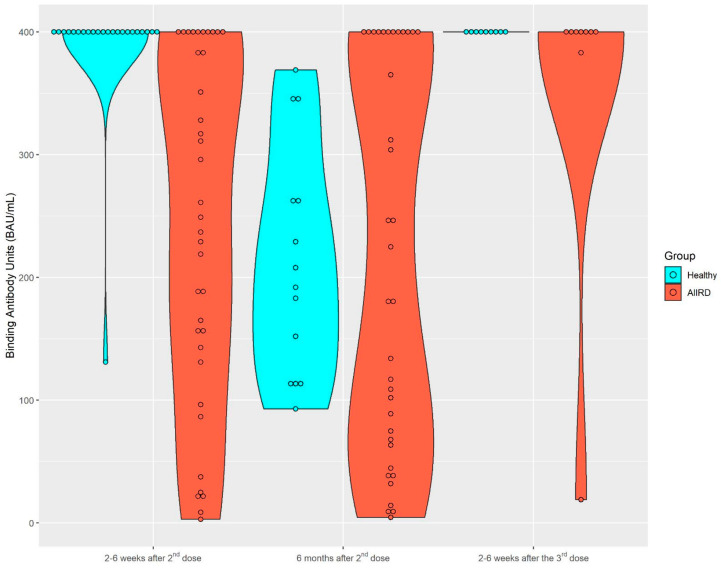
Kinetics of anti-S1/S2 IgG titers after the second BNT162b2 vaccine dose, 6 months later, and after the third dose in AIIRD patients and controls. AIIRDs, autoimmune inflammatory rheumatic diseases.

**Figure 2:**
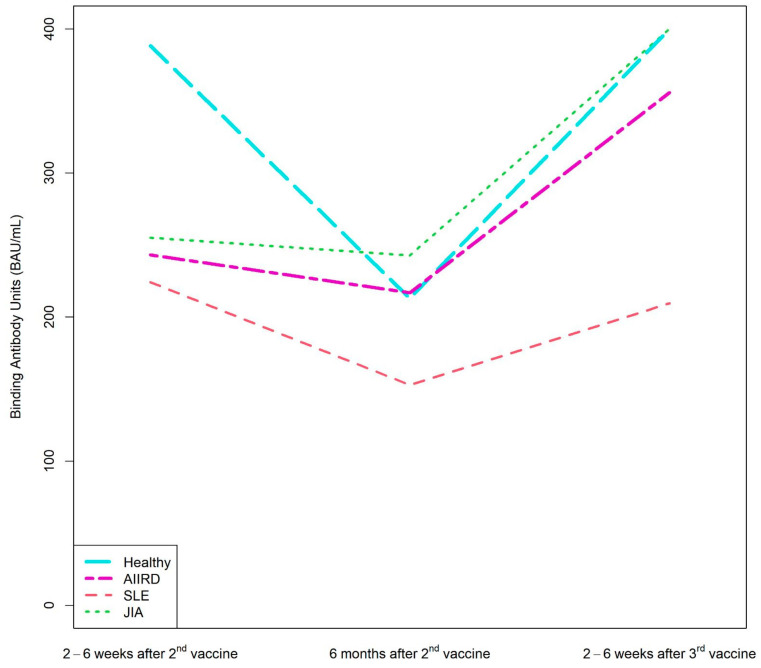
Kinetics of anti-S1/S2 IgG titers after the second BNT162b2 vaccine dose, 6 months later, and after the third dose in AIIRD patients grouped by diagnosis and in controls. AIIRDs, autoimmune inflammatory rheumatic diseases; JIA, Juvenile Idiopathic Arthritis; SLE, Systemic Lupus Erythematosus.

After the second vaccine dose, 37 patients and 23 controls provided serum samples for immunogenicity; 36 patients and 14 controls provided serum samples 6 months later. A total of nine patients and nine controls provided samples after the third vaccine. The dynamics of the S1/S2 antibody level among both study groups are presented in Figure 1 and Figure 2. Among the control group, the seropositivity rate was 100% at all three time points. Anti-S1/S2 antibody titers declined at 6 months after the second vaccine from a mean of 388.3 (SD 56.09) BAU/mL to 212.93 (SD 93.69) BAU/mL, and was restored shortly after the third vaccine dose, to 400 (SD 0) BAU/mL. As expected, the seropositivity rate was lower among AIIRD patients compared to controls, 91% at 2–8 weeks and 87% at 6 months after the second vaccination, and returned to 100% following the third dose. Anti-S1/S2 antibody titers declined at 6 months after the second vaccine from a mean of 243.1 (SD 140.8) BAU/mL to 216.9 (SD 157.6) BAU/mL and increased shortly after the third vaccine dose to 355.8 (SD 126.4) BAU/mL. In comparison to the control group, anti-S1/S2 antibody titers among the patients were significantly lower only at 2–8 weeks after the second vaccine dose (*p* < 0.0001), and there was also a wide range of antibody titers in the patient group as compared to the controls. The anti-S1/S2 antibody titers among the patients were comparable to the control group at the subsequent 6-month follow-up after the second and 2–8 weeks after the third dose. Notably, the decline of the anti-S1/S2 antibody titers within 6 months after the second vaccine dose was significantly steeper among the controls compared to patients, with a mean of −197 (SD 87.4) vs. −61.2 (SD 151.4), *p* = 0.0197, respectively. In the limited sample of controls (n = 5) and patients (n = 2) with available serologic data, the increase in the antibody titer after the third vaccine dose was comparable in both groups.

Patients with JIA maintained the highest level of anti-S1/S2 antibody at all time-points, whereas the lowest anti-S1/S2 antibody levels were found among patients with vasculitis and scleroderma (Supplementary Table S2).

Analysis of humoral response according to the use of anti-rheumatic medications showed that patients treated with csDMARDs and bDMARDs developed a comparable humoral response at all points tested. Among 17 patients treated with TNFi, those treated with TNFi monotherapy achieved numerically higher anti-S1/S2 antibody levels than those treated with combination therapy. MMF was most strongly associated with blunted humoral response to vaccination after the second vaccine dose (115 ± 89). The humoral response following the third vaccine dose evaluated in two of six patients treated with MMF was completely restored. Notably, four patients treated with anti-CD20 therapy (RTX), two patients with AAV, one patient with SLE, and one with systemic sclerosis developed an adequate seropositive response after the second vaccine dose, with the steepest decline in anti-S1/S2 antibody levels within 6 months, compared to antibody kinetics of patients treated with other medications. The humoral response following the third vaccine dose evaluated in one of these patients was completely restored. Patients treated with RTX were vaccinated 6 months after the last RTX dose and before the next dose.

### 3.4. Efficacy of the Second and Third BNT162b2 Vaccine Doses

The prevalence of COVID-19 infection observed from the second vaccine dose and forward was 48.4% (60/124) among adolescents with AIIRDs and 40% (32/80) among controls, *p* = 0.3 (Table 3). Most of the breakthrough infections (88%) occurred during the Omicron surge. During the Delta surge, the prevalence of infection was numerically higher in the patient group than in the healthy controls (n = 7, 11% vs. n = 1, 3%, *p* = 0.1555).

**Figure 3:**
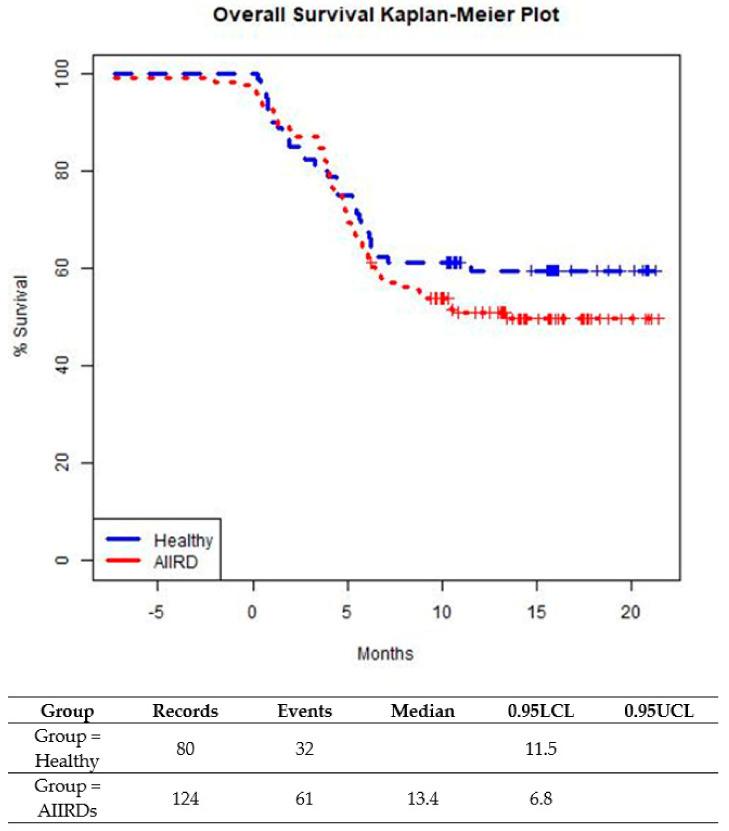
Time from last BNT162b2 vaccine dose to COVID-19 breakthrough infection in healthy adolescents and those with AIIRDs.Log-Rank *p*-value is 0.2609.

The prevalence of a breakthrough infection was similar among patients with JIA and SLE. Time-to-infection following the last vaccination was similar among AIIRD patients compared to controls until about 5 months from the last vaccination (Figure 3). After 5 months after the last vaccination, a small trend in favor of the control group was shown (*p* = 0.2609). (Figure 3). In both patient and control groups, all cases of COVID-19 infection were mild, with no severe infections or hospitalizations and no COVID-19-related complications.

## 4. Discussion

To the best of our knowledge, this prospective, multicentre, international study is the first to report the long-term safety of the BNT162b2 mRNA COVID-19 vaccine and the immunogenicity over time, induced by two and three doses of the vaccine in a cohort of adolescents with juvenile-onset AIIRDs, treated with a variety of immunomodulating agents.

The safety profile of the second and third vaccine doses was highly favorable, with most participants experiencing either no or mild side-effects and no serious side-effects in either group. Two percent of the AIIRDs cohort experienced an exacerbation of the rheumatic disease shortly after the second vaccine dose, and none of the patients who received the third vaccine reported subsequent rheumatic disease exacerbation or hospitalization.

The initial 2-dose vaccine was shown to be immunogenic in 91% of the AIIRD adolescents vs. 100% of healthy controls. However, anti-S1/S2 titers were significantly lower in the AIIRDs group, similar to reports in adults with AIIRDs [3], and there was also a large variability in the titers of the AIIRDs group.

Previous studies have shown a waning antibody response to COVID-19 vaccination in the general population [28] and AIIRDs [10,29]. In our patient and control groups, we observed a waning of the anti-S1/S2 antibody titers over 6 months, with a faster decline in the control group compared to patients. This finding needs further clarification in larger trials.

Following the third vaccine, the seropositivity rate increased to 100% in adolescents with AIIRDs. Furthermore, anti-S1/S2 titers reached a higher level as compared to those measured following the 2-dose vaccine, supporting the recommended booster vaccine dose in adolescents with AIIRDs and also consistent with the response observed in adults with AIIRDs. Notably, in the largest cohort of AIIRD adults, seropositivity after the third vaccine (82.3%) increased to a rate similar to that documented after the second dose (84.7%) [10]. The more vigorous response to the third dose in our adolescent patient cohort compared to adult patients may be explained by a stronger immunogenic response at younger ages and/or the more prevalent use of combination anti-rheumatic therapy in the adult cohort.

A meta-analysis published in 2022 showed that pooled seroconversion rates after a two-dose SARS-CoV-2 vaccination regimen were lower in patients with immune-mediated inflammatory disease compared to healthy controls. Importantly, certain therapies did not impact the seroconversion rates, including anti-TNF, anti-integrin, anti-IL-17, anti-IL6, and anti-12/23, while other therapies resulted in poorer responses [30], including anti-CD20 and anti-CTLA-4. The current study was not large enough to determine the impact of the anti-rheumatic medication on the humoral response to vaccination. We reported an adequate humoral response in patients treated with methotrexate and anti-TNF and a blunted humoral response after the second vaccine dose in a small group of patients treated with MMF, which is consistent with a previous report [3]. According to the literature regarding adults with AIIRDs, RTX is a dominant factor for the lack of antibody response to the COVID-19 vaccine at all time points, especially among patients treated with RTX within 6 months prior to vaccination. B-cell depletion also consistently correlates with negative immunological response [31,32]. Our small cohort of adolescents treated with RTX had a good humoral response. This might be because the vaccines were given at the appropriate times or because of the favorable immunogenic response at young ages.

It is important to note that there is still no consensus regarding the correlation between anti-spike antibody levels and protection from COVID-19 infection. Vaccination against COVID-19 induces both humoral and cellular responses, but it is widely thought that vaccine-induced neutralizing antibodies to the receptor binding domain of the SARS-CoV-2 S protein are a plausible mechanism of protection. A few studies [33,34,35] in healthy adults demonstrated a significant correlation between neutralizing antibody titers and vaccine efficacy, with most of the breakthrough infections reported as mild or asymptomatic [35]. Sakir et al. studied 630 patients with AIIRDs and reported an association between breakthrough infections and seronegativity following a COVID-19 vaccination [36]. This provides a basis for exploring postvaccination antibody titers as a potential predictor of breakthrough infection in patients with AIIRDs.

The breakthrough infection rate in this study was relatively similar between the AIIRDs and control groups, with a slightly higher rate for the AIIRDs group. Survival analysis also showed very similar curves for both groups in the first 5 months from the last vaccination, and afterward, the control group showed a favored, not statistically significant trend. Most of the breakthrough COVID-19 infections occurred either before or after the third vaccine. We suggest this as a means to estimate the actual protection from the vaccines. Most cases (88%) were documented during the Omicron surge, which is not surprising, as it is now evident that the original COVID-19 vaccine did not provide enough protection from the Omicron variant [37,38,39]. It is notable that during the Delta surge, the breakthrough infection rates were low, similar to a previous report in adolescents with AIIRDs [40]. Even though the COVID-19 infection rates during the Delta and Omicron waves were higher among the AIIRD patients as compared to the healthy controls, the differences were not statistically significant.

These data are encouraging to the community of adolescents with juvenile-onset AIIRDs and can help reduce hesitancy to vaccinations. The finding that no severe COVID-19 cases were documented can be explained by the effectiveness of the vaccine, that most of the young population experienced a mild form of the disease. In addition, the Omicron variant mostly caused mild illness [41,42].

There were several limitations to this study. The number of participants who provided serum samples for humoral response evaluation at 6 months and following the third vaccine decreased significantly, despite the ongoing COVID-19 pandemic. This is probably because the population was less compliant with the third vaccine dose.

In addition, due to the relatively small sample size, and specifically the diversity of rheumatic diseases and medications included in this juvenile-onset cohort, it was not possible to investigate the impact of immunomodulatory medications and type of disease on the anti-S1/S2 titers. In addition, the matching by age was not optimal, as the control group was younger than the patient group, with mean ages of 13.7 and 15.5 years, respectively. However, this might actually strengthen our findings of high seropositivity rates, as younger control children could have more robust immune activity.

## 5. Conclusions

To summarize, this is the first longitudinal study of adolescents with AIIRDs to report long-term safety, immunogenicity dynamics, and breakthrough COVID-19 infections after the second and third doses of the BNT162b2 mRNA COVID-19 vaccine. We found an excellent safety profile with minimal to no side effects after the vaccinations overall and minimal risk of rheumatic disease exacerbation. In addition, the rates of breakthrough COVID-19 infections, and the time from the last vaccination to infection were similar between groups. All the healthy controls and most of the adolescents with AIIRDs were seropositive following the second vaccine, followed by a decline in anti-spike S1/S2 antibody titers in both groups over the 6 month period after the second vaccine. The titers were restored in all patients and controls after the third vaccine dose.

The findings of this study can help decrease vaccine hesitancy in adolescents with juvenile-onset AIIRDs. Based on our results, it is recommended to booster adolescents with rheumatic disease with the BNT162b2 mRNA COVID-19 vaccine. Additional studies with larger numbers of adolescents with AIIRDs are needed to evaluate the impact of anti-rheumatic/immunomodulatory therapies on the long-term immune response to the BNT162B2 vaccine. 

## Figures and Tables

**Table 1:** Baseline characteristics of adolescents with AIIRDs and controls.

Variable	AIIRDs (124)	Controls (80)	*p*-Value
Demographics			
Age, mean (SD)	15.48 (1.99)	13.74 (1.36)	<0.0001
Female, N (%)	74 (60%)	41 (51%)	0.2682
Country			
Israel, N (%)	97 (78%)	80 (100%)	<0.0001
Slovenia, N (%)	27 (22%)		
Disease duration, mean (SD)	4.12 (3.79)		
AIIRDs, N (%)	124 (100%)		
JIA, N (%)	54 (44%)		
SLE, N (%)	20 (16%)		
Autoinflammatory diseases, N (%)	11 (9%)		
Scleroderma, N (%)	7 (6%)		
Vasculitis, N (%)	6 (5%)		
Uveitis, N (%)	8 (6%)		
Myositis, N (%)	5 (4%)		
IBD-related arthritis, N (%)	7 (6%)		
Other, N (%)	6 (5%)		
Immunosuppressive treatment, N (%)	92 (74%)		
Methotrexate treatment, N (%)	27 (22%)		
Biologic treatment, N (%)	48 (39%)		

AIIRDs, autoimmune inflammatory rheumatic diseases; JIA juvenile idiopathic arthritis; SLE, systemic lupus erythematosus; IBD, inflammatory bowel diseases.

**Table 2:** Adverse events following BNT162b2 mRNA COVID-19 vaccine among adolescents with AIIRDs and controls.

Variable	2-Weeks after 1st Vaccination			2–8 Weeks after 2nd Vaccination			2–8 Weeks after 3rd Vaccination		
	AIIRDs (n = 124)	Controls (n = 80)	*p* Value	AIIRDs (n = 124)	Controls (n = 80)	*p* Value	AIIRDs (n = 64)	Controls (n = 30)	*p* Value
Local reactions	88 (71%)	44 (55%)	0.03	89 (72%)	44 (55%)	0.02	25 (39%)	22 (73%)	0.004
Systemic reactions, n (%)									
Fever	6 (5%)	2 (2%)	0.64	18 (15%)	8 (10%)	0.47	7 (11%)	1 (3%)	0.40
Chills	5 (4%)	2 (2%)	0.84	17 (14%)	3 (4%)	0.04	4 (6%)	1 (3%)	0.92
Runny nose	4 (3%)			6 (5%)	1 (1%)	0.32	1 (2%)		
Cough	3 (2%)			5 (4%)	1 (1%)	0.46	1 (2%)		
Nausea	5 (4%)	2 (2%)	0.84	6 (5%)	2 (2%)	0.63	3 (5%)		
Vomiting	1 (1%)	2 (2%)	0.69	3 (2%)	2 (2%)	1	0 (0%)		
Fatigue/weakness	14 (11%)	6 (8%)	0.51	33 (27%)	12 (15%)	0.07	9 (14%)	2 (7%)	0.48
Myalgia	12 (10%)	6 (8%)	0.77	23 (19%)	7 (9%)	0.08	6 (9%)	2 (7%)	0.96
Arthralgia	7 (6%)	1 (1%)	0.22	14 (11%)	2 (2%)	0.04	2 (3%)		
Headache	10 (8%)	8 (10%)	0.82	29 (23%)	9 (11%)	0.04	8 (12%)	2 (7%)	0.61
Chest pain	0 (0%)			0 (0%)			3 (2%)		
Pericarditis or myocarditis	0 (0%)			0 (0%)			0 (0%)		
Herpes zoster	0 (0%)			0 (0%)			0 (0%)		
Allergic reaction	0 (0%)	1 (1%)	0.82	0 (0%)			0 (0%)		
Exacerbation of rheumatic disease symptoms	5 (4%)			3 (2%)			0 (0%)		
Hospitalization within 2 weeks post-vaccine	2 (2%)			0 (0%)			0 (0%)		

AIIRDs, autoimmune inflammatory rheumatic diseases.

**Table 3:** COVID-19 infection events following vaccination among patients with AIIRDs compared with controls.

Group	Total	*p*-Value	Between 2nd and 3rd Doses	*p*-Value	After 3rd Dose	*p*-Value
Controls	80		80		30	
	32 (40%)		14 (17.5%)		18 (60%)	
AIIRD patients	124		124		64	
	60 (48.4)	0.3024	33 (26.6%)	0.1806	27 (42.2%)	0.1645
JIA	54		54		23	
	26 (48.15)	0.4496	17 (31.5%)	0.0942	9 (39.1%)	0.219
SLE	20		20		10	
	7 (35%)	0.8778	3 (15%)	1	4 (40%)	0.463

AIIRDs, autoimmune inflammatory rheumatic diseases; JIA, Juvenile Idiopathic Arthritis; SLE, Systemic Lupus Erythematosus.

## Data Availability

All the data relevant to this study are included in the manuscript, tables, and figures. Additional data will be provided upon request.

## Supplementary Materials

The following supporting information can be downloaded at: https://www.mdpi.com/article/10.3390/vaccines11040819/s1, Table S1: Treatments per diagnosis among adolescents with AIIRD; Table S2: Anti-S1/S2 IgG titres following the second and third BNT162b2 vaccine dose in adolescents with AIIRD grouped by disease type and controls.
