# Supplementary material for: Safety and Immunogenicity Following the Second and Third Doses of the BNT162b2 mRNA COVID-19 Vaccine in Adolescents with Juvenile-Onset Autoimmune Inflammatory Rheumatic Diseases: A Prospective Multicentre Study
Source: Vaccines (Basel). 2023 Apr 10;11(4):819. doi: 10.3390/vaccines11040819 (PMC10145688; doi:10.3390/vaccines11040819)
Supplement: Supplementary file 1 [file vaccines-11-00819-s001.zip › vaccines-2294133-supplementary.pdf]

SUPPLEMENTARY FILE

Table S1. Treatments per diagnosis among adolescents with AIIRD

**AIIRD diagnosis**	**csDMARDS**	**bDMARDS**	**MTX**	**MMF**	**TNFi**	**IL6i**	**Anti-CD20**	**Steroids**	**Plaquenil**	**JAKi**
All AIIRD, N=124 (%)	57 (46.0)	48 (38.7)	27 (21.8)	13 (10.5)	39 (31.5)	7 (5.7)	4 (3.2)	21 (16.9)	26 (21.0)	5 (4.0)
JIA, n=54 (%)	24 (44.4)	26 (48.2)	19 (35.2)	1 (1.9)	23 (42.6)	6 (11.1)	0 (0)	3 (5.6)	0 (0)	4 (7.4)
SLE (n=20)	20 (100)	1 (5)	0 (0)	8 (40)	0 (0)	0 (0)	1 (5)	11 (6)	20 (100)	0
Autoinflammatory diseases (n=11)	1 (9.1)	3 (27.3)	0 (0)	0 (0)	2 (18.2)	0 (0)	0 (0)	0 (0)	1 (9.1)	0
Scleroderma (n=7)	4 (57.1)	1 (14.3)	2 (28.6)	4 (57.1)	0	0	1 (14.3)	1 (14.3)	2 (28.6)	0
Vasculitis (n=6)	0	3 (50)	0	0	1 (16.7)	0 (0)	2 (33.3)	1 (16.7)	0)	0
Uveitis (n=8)	3 (37.5)	5 (62.5)	3 (37.5)	0	5 (62.5)	1 (12.5)	0	2 (3)	0)	0
Myositis (n=5)	3 (60)	0	2 (40)	0	0	0	0	2 (40)	3 (60)	1 (20)
IBD-related arthritis (n=7)	1 (14.3)	7 (100)	1 (14.3)	0	7 (100)	0	0	0	0	0

AIIRD, autoimmune inflammatory rheumatic disease; csDMARDs, conventional synthetic disease modifying antirheumatic drugs; bDMARDs, biologic disease modifying antirheumatic drugs; MTX, methotrexate; MMF, mycophenolate mofetil; TNFi, tumour necrosis factor α inhibitors; IL, interleukin; JAKi, janus kinase inhibitors; JIA, juvenile idiopathic arthritis; SLE, systemic lupus erythematosus, IBD, inflammatory bowel disease.

Table S2. Anti-S1/S2 IgG titres following the second and third BNT162b2 vaccine dose in adolescents with AIIRD grouped by disease type and controls

Immuno-modulatory treatment, N	6 weeks after vaccination		6 months after 2nd vaccination		After 3rd vaccination	
	Seropositivity rate, n (%)	Anti S1/S2 IgG titre, mean ± SD (BAU/ml)	Seropositivity rate, n (%)	Anti S1/S2 IgG titre, mean ± SD (BAU/ml)	Seropositivity rate, n (%)	Anti S1/S2 IgG titre, mean ± SD (BAU/ml)
Healthy, n=23	23 (100)	388.3 (561)	14 (100)	212.93 (93.7)	9 (100)	400 (0)
AIIRD, n=45	41 (91)	243.09 (140.84)	41 (87)	216.91 (157.6)	13 (100)	355.78 (126.4)
Vasculitis, n=4	3 (75)	218.15 (203.17)	3 (75)	129.8 (182.41)	1 (100)	383 (NA)
SLE, n=8	8 (100)	224.09 (161.66)	8 (89)	152.77 (149.16)	2 (100)	209.5 (269.41)
Myositis, n=1	1 (100)	187 (NA)	2 (100)	400 (0)	0 (0)	NaN (NA)
Scleroderma, n=4	3 (75)	189.33 (191.75)	1 (50)	109 (NA)	2 (100)	400 (NA)
JIA, n=15	14 (93)	255.04 (142.8)	20 (87)	242.75 (164.22)	5 (100)	400 (0)
Uveitis, n=3	3 (100)	250 (137.15)	0 (0)	NaN (NA)	2 (100)	400 (0)
IBD, n=6	6 (100)	250.77 (126.03)	1 (100)	89 (NA)	0 (0)	NaN (NA)
Autoinflammatory diseases, n=3	2 (67)	312 (22.63)	4 (100)	297.67 (88.62)	0 (NaN)	NaN (NA)
Other, n=1	1 (100)	400 (NA)	2 (100)	312.5 (123.74)	1 (100%)	400 (NA)

AIIRD, autoimmune inflammatory rheumatic disease; SLE, systemic lupus erythematosus, JIA, juvenile idiopathic arthritis; IBD, inflammatory bowel disease.
